# Magnetic Fields Impact on PIN‐FORMED Protein Polarity in *Arabidopsis thaliana*

**DOI:** 10.1111/ppl.70274

**Published:** 2025-05-23

**Authors:** Azita Shabrangy, Christian Luschnig

**Affiliations:** ^1^ Molecular Systems Biology (MOSYS), Department of Functional and Evolutionary Ecology, Faculty of Life Sciences University of Vienna Vienna Austria; ^2^ Institute of Molecular Plant Biology (IMPB) BOKU University Wien Austria

**Keywords:** *Arabidopsis thaliana*, gradient magnetic fields, plant growth, polar auxin transport, root development, static magnetic fields

## Abstract

Magnetic fields (MF) exert a considerable influence on biological processes in various organisms, including prominent effects on plant growth and development. Plant responses to MFs are highly diverse, implying that a plethora of processes are affected, hampering their molecular characterization. In this report, we employed the well‐characterized plant model *Arabidopsis thaliana* to determine root growth responses to both static magnetic fields (SMF) and gradient magnetic fields (Gradient MF). SMF exposure resulted in a dosage‐dependent inhibition of root elongation growth and altered auxin‐responsive reporter expression, whilst Gradient MF exposure interfered with root curvature and auxin signaling under conditions of minimized gravity effects. Mutants deficient in components of the *Arabidopsis* auxin transport machinery were less responsive to MFs than wild type, indicative of MF‐induced effects on polar auxin transport. When viewing the subcellular localization of PIN‐FORMED (PIN) auxin efflux transporters in root meristems, we found that MFs impact their subcellular distribution in root cap and epidermis cells. These effects on PIN localization hint at a molecular switch, linking cellular protein dynamics, auxin transport, and morphogenesis, by which MFs impact the growth of higher plants.

## 1 Introduction

The geomagnetic field (GMF) constitutes an indispensable characteristic of our natural habitat, shielding Earth from particle radiation, like that emanating from solar winds. The strength of the vertical component of the GMF varies, ranging from 67 μT at the magnetic poles to zero at the magnetic equator on the Earth's surface. Conversely, the horizontal component peaks at approximately 33 μT at the magnetic equator and diminishes to zero at the magnetic poles (Valiron et al. 2005; Maffei 2014). A multitude of organisms, spanning from bacteria to vertebrates, utilize the GMF for orientation and navigation. Notably, plants, which exhibit sensitivity to various stimuli, including light, gravity, or touch, also respond to magnetic fields (MF), yet the significance of such responses remains vague (Kiss 2000; Valiron et al. 2005; Yan et al. 2012; Hasenstein et al. 2013; Maffei 2014).

Reports describe plant responses to MFs, with typically higher field strength than the GMF. The application of MFs within a range of 1 mT to several 100 mT during early vegetative growth stages was repeatedly found to enhance crops' growth and yield (Shine et al. 2011; Anand et al. 2012; Bilalis et al. 2012; Payez et al. 2013; da Silva and Dobranszki 2016), while further reports documented inhibitory MF effects (Maffei 2014).

Employing the model organism *Arabidopsis thaliana* revealed some physiological insights into MF effects. Strikingly, reduced *Arabidopsis* growth was detected under near‐null‐magnetic‐field (NNMF) conditions, underscoring the necessity of the GMF for plant development (Xu et al. 2018). On the other hand, weak SMFs ranging from NNMF to 122 μT affect several developmental responses, including cryptochrome‐independent seed germination, phytochrome and cryptochrome‐dependent hypocotyl elongation, and photo‐accumulation of anthocyanins as well as chlorophylls (Dhiman et al. 2023). A reversal of GMF polarity, generated by octagonal coils for each of the spatial axes, was found to significantly inhibit seedling growth and to modulate gene expression, supporting scenarios with GMF influencing plant evolution in geological timescales (Bertea et al. 2015). Meticulously designed magnetic gradients were found to modulate the directional growth of plant organs, a response termed magnetotropism (Kuznetsov and Hasenstein 1996; Nechitailo et al. 2001; Zhou et al. 2024). Such magnetic gradients facilitate the movement of diamagnetic compounds, including gravity‐sensing statoliths, and were therefore suggested to influence directional organ growth (Hasenstein et al. 2013). Despite all these astounding insights into plants' responses to MFs, our understanding of mechanisms involved at a molecular level remains vague (Hafeez et al. 2023).

Evidently, cellular components will exert variable responses to MF application, simply as a result of their differing dia‐ and paramagnetic properties (Occhipinti et al. 2014; Shokrollahi et al. 2018). The diversity of compounds and structures potentially responsive to MFs likely accounts for the numerous molecular processes affected, ranging from subtle adjustments in enzyme activities (Haghighat et al. 2014) to major modifications in plants' transcriptomes and proteomes (Paul et al. 2006; Paponov et al. 2021; Shabrangy et al. 2021). *Arabidopsis* seedlings exposed to NNMF conditions responded with differential, frequently opposing, root and shoot transcriptome patterns, with a large number of stress‐related genes being affected (Parmagnani et al. 2022). Furthermore, variations in MF strength caused differing responses at the transcriptome level, indicative of dosage‐dependent effects (Paponov et al. 2021). Genes implicated in the control of auxin responses as well as auxin homeostasis are prominently represented among loci that respond to MF variations (Xu et al. 2018; Zotti et al. 2018; Jin et al. 2019). Accordingly, it was suggested that adjustments in signaling triggered by the phytohormone auxin would participate in MF‐induced growth responses (Paponov et al. 2021; Zhou et al. 2024). Along these lines, Jin and colleagues provided evidence for SMF exposure impacting the expression of auxin uptake and auxin efflux carriers, which might modulate polar auxin transport (PAT) and, consequently, plant morphogenesis (Jin et al. 2019).

In this study, we aimed at an in‐depth characterization of the potential contribution of the *Arabidopsis* auxin transport machinery to MF‐induced adjustments in plant growth responses. For this purpose, we evaluated root elongation growth in homogeneous SMFs with the MF direction perpendicular to the gravity vector. Furthermore, we determined root curvature in a gradient MF with a (magnetic) South pole‐seedling‐South pole setup upon rotation on a clinostat, thereby mitigating the unilateral influence of gravity. Auxin‐related effects were determined by analyses of root growth in wild type and auxin‐transport mutants, combined with an analysis of auxin‐responsive reporters and auxin‐transport proteins. Emphasis was given to the dynamics of PIN protein distribution in response to SMF and Gradient MF growth conditions, establishing MFs as effectors of PIN localization at the plasma membrane.

## 2 Materials and Methods

### 2.1 Plant Material and Growth Conditions

The *Arabidopsis thaliana* mutants utilized in this study, namely *aux1‐7* (Pickett et al. 1990), *eir1‐4* (Abas et al. 2006), *eir1‐1* (Roman et al. 1995), *pin3‐5 pin4‐3 pin7‐1* (Blilou et al. 2005) and complemented *eir1‐4 PIN2::PIN2:VEN* (Leitner et al. 2012) have been described previously. Wild type Col‐0 was used as a control for all the growth experiments. *AtIAA2::GUS* (Luschnig et al. 1998), *DR5rev::3XVENUS‐N7* (Heisler et al. 2005), *PIN2::PIN2:VEN* (Leitner et al. 2012), and *PIN3::PIN3:YFP* (Zadnikova et al. 2010) reporter lines have been used to assess MF effects on auxin distribution. Seeds were sterilized using a 6% (v/v) hypochlorite solution and 0.05% Triton X‐100 for 5 min, followed by immersion in 70% (v/v) ethanol for 3 min, and subsequent triple rinsing with sterile distilled water. They were then sown in a line at the center of petri dishes containing Murashige and Skoog (MS) basal medium (Murashige and Skoog 1962) supplemented with 1% plant agar and 1% (w/v) sucrose. Following a 2‐day storage period at 4°C, the seeds were cultivated at 22°C ± 1°C, with a photoperiod of 16 h light and 8 h dark. Root lengths as well as the direction of root growth were determined by scanning the seedlings using a flatbed scanner to acquire images suitable for quantification via ImageJ (https://imagej.net/ij/). Datasets obtained from growth assays and microscopy were evaluated as described for the individual experiments.

### 2.2 Setup of Homogenous SMFs

**FIGURE 1:**
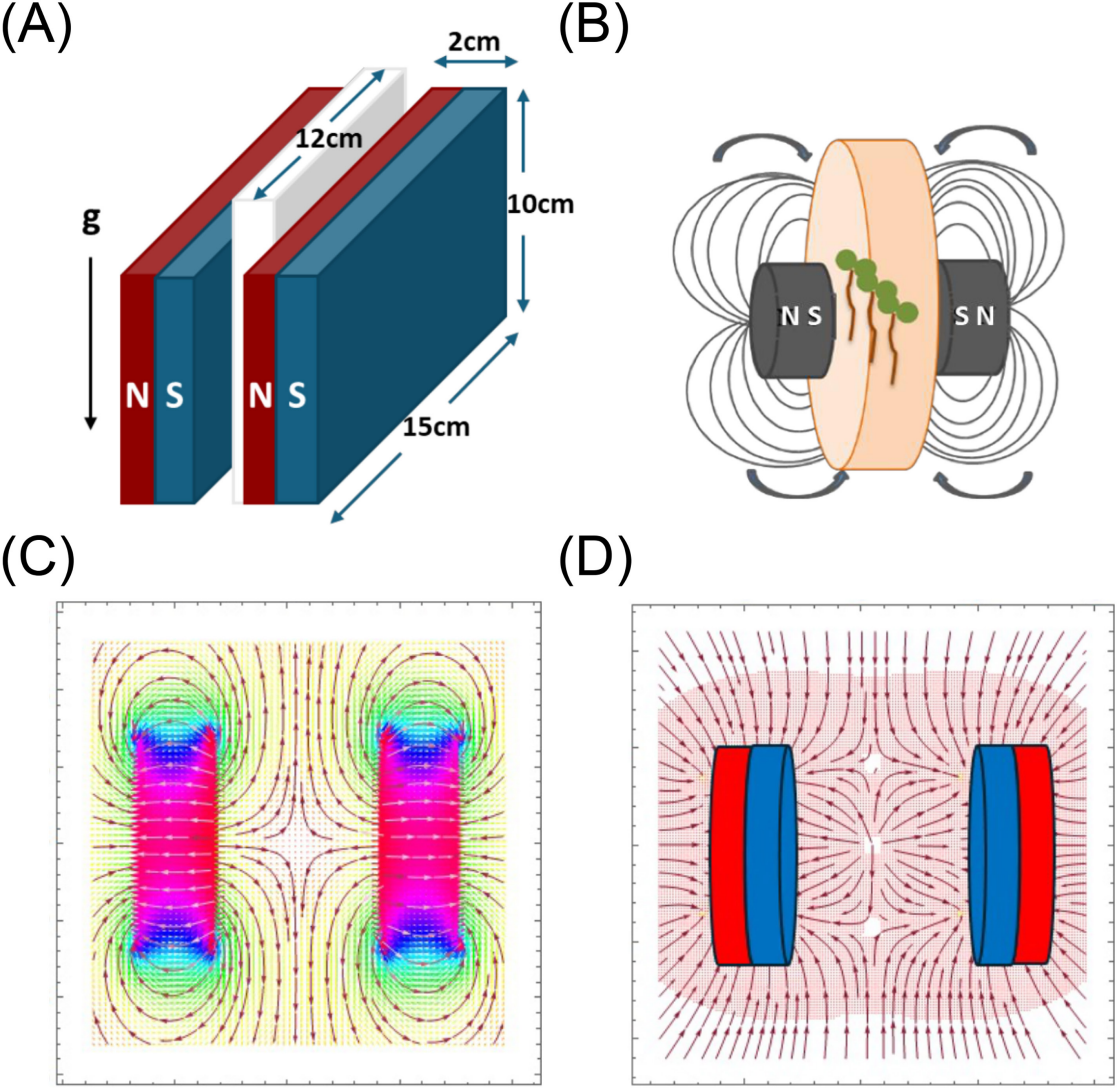
Setup of MF conditions. (A) An SMF intensity of 127 mT was generated in the central area between two magnets separated by a distance equivalent to the thickness of a petri dish. This produced a uniform field, with the field direction running from the north (N) to the south (S) pole, perpendicular to the gravitational vector. (B) A Gradient MF was generated using two small circular magnets arranged in a South‐to‐South pole orientation, with samples positioned between them and incubated on a clinostat. (C) MF distribution between two pole‐to‐pole opposed magnets (length 7 mm, diameter 14 mm, gap 15 mm); curves with arrows represent MF lines. (D) MF gradient distribution; arrows show the directions of the magnetic gradient force in the central region between the magnets.

We generated homogeneous SMFs for continuous exposure of seedlings between two ferrite magnet slabs (150 mm × 20 mm × 120 mm each) in the presence of the local GMF. The MF orientation was aligned perpendicular to the gravity vector (Figure 1A). The uniformity and accuracy of the magnetic flux densities were calibrated using a Gauss‐meter with a Hall Effect‐based B‐probe (HIRS Magnetic Instruments Ltd., Type GM08), ensuring homogeneous fields across the petri plates and along the z‐axis, where 15–20 seedlings were centrally positioned. For this, *Arabidopsis* seeds were positioned on vertically oriented square nutrient plates (Greiner, 120 × 120 × 17 mm), sandwiched between the two large ferrite magnet slabs (Figure 1A), with control setups established using dark wooden blocks (150 mm × 20 mm × 120 mm). In this setup, seedlings were illuminated from the top. For setups using differing field strengths, magnets were separated by additional clear polystyrene petri dishes, with distances ranging from 17 to 153 mm, and SMF intensities determined using a Gauss‐meter, as detailed in Table 1.

**TABLE 1:** SMF intensity produced by two magnets (150 × 20 × 120 mm), at distances as indicated.

Distance of magnets (mm)	17	51	85	153
Intensity of SMF (mT)	127	103	89	60

### 2.3 Setup of Gradient MFs

This setup allowed for the determination of the directional root growth of *Arabidopsis* wild type and mutants exposed to a Gradient MF. A clinostat, operating at a rotational speed of 0.07 rpm in a clockwise direction, was utilized to reduce gravity effects, allowing MFs to impose their effects. In the setup, 4‐day‐old *Arabidopsis* seedlings grown on MS medium were exposed to Gradient MFs for 24 h. For this purpose, two cylindrical neodymium magnets (300 mT flux density, 14 mm diameter, 7 mm height) were arranged on the top and bottom sides of round nutrient plates (50 mm diameter, 15 mm height), over a central area where the roots of the seedlings were positioned. We employed a repelling MF geometry with the South poles of both magnets facing each other (Figure 1B). MF lines in this setup are indicated as arrows (Figure 1C), as is the magnetic gradient force distribution in the central region between the two pole‐to‐pole aligned magnets (Figure 1D) (Zablotskii et al. 2016). Gradient MF‐exposed and control plates (nutrient plates, lacking magnets) were positioned at the edge of the rotating wheel on the clinostat (diameter 250 mm), with the root tips pointing towards the center of the rotating wheel. Petri dishes were covered with light impermeable non‐magnetic foil during the treatment, and root curvature was determined by scanning the seedlings using a flatbed scanner followed by quantification using ImageJ (http://rsb.info.nih.gov/ij/). Each experiment involved measuring seedlings across three independent repeats.

### 2.4 Microscopy and Sample Preparation

After treatment, seedlings were immediately mounted and observed on a Leica SP5 confocal laser scanning microscope (CLSM). YFP/Venus was excited at 514 nm (detection from 525 to 578 nm). Fluorescence signals were processed using Leica software LAS AF 3.1 or ImageJ. Two to three biological repeats with intrinsic technical repeats were performed, and identical settings were used for image acquisition. For PIN2‐Venus, signal intensities were quantified at apical and lateral sides of root meristem epidermis cells in the transition zone, with grey values from the intracellular area used for normalization. For PIN3‐YFP polarity assessment, six‐day‐old *PIN3::PIN3:YFP* seedlings, grown vertically under a 16/8‐h light/dark cycle at 22°C, were transferred onto small nutrient plates and placed on a clinostat. The seedlings were rotated for 90–120 min, with their roots facing the center (rotation axis) of the clinostat. Control plates and plates with magnets attached above and below the roots in a South‐to‐South orientation were used. After incubation, seedlings were transferred onto slides and immediately used for CLSM. As a control for clinostat effects, seedlings that remained undisturbed on vertically oriented nutrient plates were used. The data represent the ratio of YFP signals at the basal side versus the outer lateral sides of tier 2 and tier 3 root cap columella cells. A similar setup has been used for the analysis of signals in *DR5rev::3XVENUS‐N7* root meristems, as described by Su et al. (2023). For studying the effects of Gradient MF conditions on the distribution of statoliths in the root cap, 5‐day‐old wild type seedlings were clinorotated for 2 h, followed by immediate analysis at the microscope.

For GUS‐staining, seedlings were immersed in a staining buffer (sodium phosphate 0.1 M, EDTA 10 mM, K_3_FeCN_6_ 0.5 mM, K_4_FeCN_6_ 0.5 mM, Triton X 100 0.1% (v/v), pH 7.0, and X‐Gluc 0.1 mM) in darkness at 37°C. Subsequently, the seedlings were clarified in a solution of acetic acid/ethanol (1:3 v/v) for 2 h and subjected to a series of graded ethanol concentrations. The seedlings were then mounted in 50% Glycerol. Image acquisition was conducted using a light microscope (Leica DM 5500) equipped with a DFC 300 FX camera (Leica).

## 3 Results

### 3.1 SMFs Exhibit Dosage‐Dependent Inhibitory Effects on *Arabidopsis* Root Elongation

**FIGURE 2:**
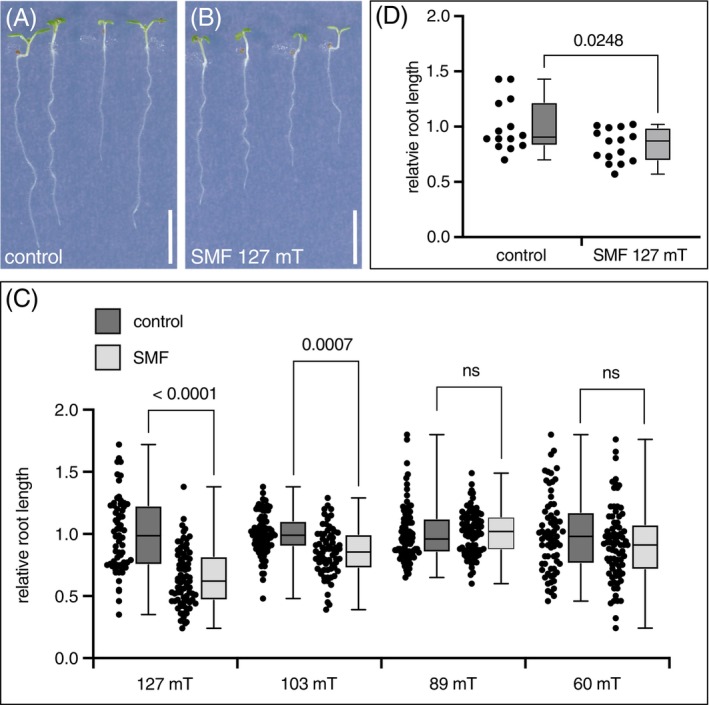
SMF incubation modulates root elongation in a dosage‐dependent fashion. (A) 6‐day‐old wild type seedlings grown on vertically oriented nutrient plate flanked by wooden blocks to ensure uniform light conditions for control and treated samples. (B) 6‐day‐old wild type seedlings grown on vertically oriented nutrient plate flanked by cuboid ferrite magnets producing a field of 127 mT. (C) Relative root length of 6‐day‐old wild type seedlings under control conditions or exposed to SMF conditions of different strength (127, 103, 89, 60 mT). A total of 57 to 93 seedlings was examined for each condition in 3 biological repeats. (D) Relative root length of wild type seedlings grown on 1% (w/v) agar plates under control conditions (*n* = 14) and exposed to an SMF (*n* = 15). Individual root lengths were normalized to the average root length of the individual control samples, followed by one‐way ANOVA and Tukey's HSD post hoc test. Second/third quartiles and *p*‐values are indicated; ns, not significant. Size bars: (A, B) = 5 mm.

To test for possible effects of MFs on the *Arabidopsis* PAT machinery in roots, we first had to establish growth conditions, resulting in reproducible root phenotypic traits. A number of different experimental setups have been reported to impact germination and further growth of plants (Maffei 2014; Sarraf et al. 2020; Hafeez et al. 2023). In one of the tested setups, we positioned *Arabidopsis* seeds on vertically oriented nutrient plates, sandwiched between two large ferrite magnet slabs (Figure 1A). Under these conditions, the field lines were approximately perpendicular to the gravity vector and hence the seedlings' growth axis. For the control setup, we made use of oak wooden blocks with exactly the same dimensions as the magnets employed. Upon germination and incubation of wild type in such homogeneous SMFs for 6 days, we did not observe any deviations in the directionality of primary root growth. However, we detected a significant inhibition of primary root elongation when applying a SMF with an intensity of 127 mT (Figure 2A–C). When further testing MF effects on root growth, we found that growth inhibition exhibited a correlation with the flux density of the SMF, as seedlings exposed to SMF with an intensity of 127 mT displayed the strongest reduction in root length compared to the controls, and root growth inhibition was also observed in seedlings exposed to 103 mT for 6 days (Figure 2B). In contrast, exposure of wild type to 89 mT or 60 mT for 6 days did not result in significant alterations in root growth (Figure 2C). In related experiments, we asked whether MF effects on the growth medium could indirectly impact seedling growth by using a prepared a medium containing only 1% (w/v) plant agar but lacking all micro‐ and macro‐nutrients. After 6 days exposure to 127 mT, a root growth inhibition was detectable on SMF‐exposed agar plates lacking nutrients. These differences, however, were less pronounced than those observed on nutrient plates. Therefore, the contribution of nutrient effects under SMF conditions cannot be categorically excluded (Figure 2D).

In conclusion, setting up growth conditions revealed a dosage‐dependent inhibitory effect of SMFs on root elongation when oriented in a perpendicular orientation to the gravity vector. For further assessment of MF effects on the PAT machinery, we therefore pursued this setup with a field strength of 127 mT.

### 3.2 Root Growth Inhibitory SMF Effects Require a Functional Auxin Transport Machinery

**FIGURE 3:**
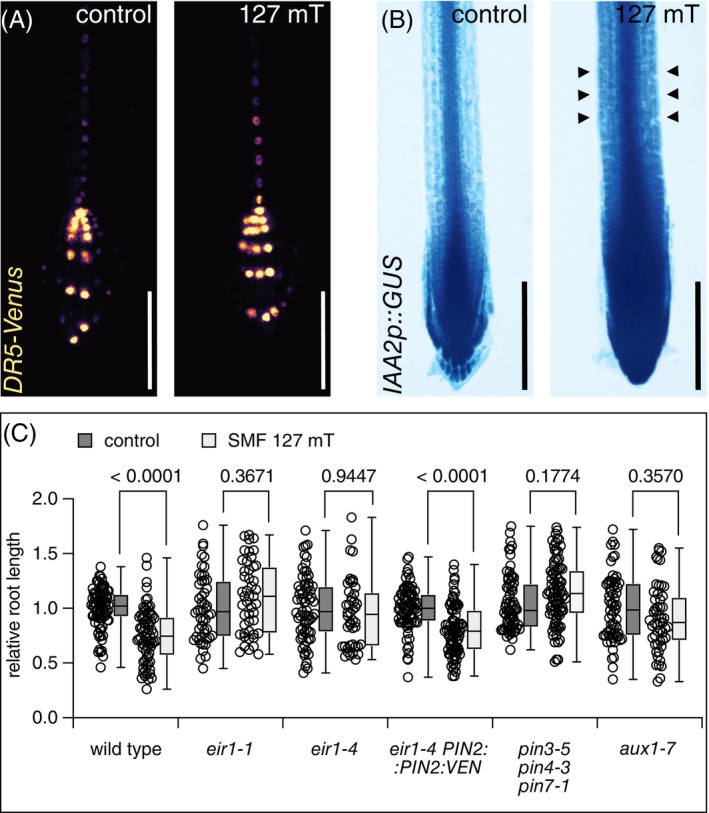
SMF‐mediated inhibition of root elongation coincides with altered expression of an auxin reporter and requires elements of the auxin transport machinery. (A) *DR5rev::3XVENUS‐N7* signals in root meristems under control conditions (left) and after 6 days exposure under SMF conditions (right). (B) *AtIAA2p::GUS* activity in root meristems under control conditions (left) and after 6 days exposure under SMF conditions (right). Arrowheads: Elevated GUS‐staining in the root meristem elongation zone. (C) Relative root length of 6‐day‐old wild type, *eir1‐1*, *eir1‐4*, *eir1‐4 PIN2::PIN2:VEN*, *pin3‐5 pin4‐3 pin7‐1*, and *aux1‐7* seedlings grown under control conditions or exposed to 127 mT SMFs. A total of 47 to 85 individuals was analyzed for each genotype and condition in 2 biological repeats. Second/third quartiles and *p*‐values are indicated. Size bars: (A) = 50 μm, (B) = 100 μm.

In order to test for MF‐induced responses in auxin signaling and/or distribution, we first analyzed the expression of auxin‐responsive reporter lines. No striking alterations in reporter signals in the root cap of *DR5rev::3XVENUS‐N7* seedlings were observed after 6 days of SMF exposure (Figure 3A), indicating that auxin distribution and/or signaling is not affected in these cells. We also tested auxin‐responsive *AtIAA2p::GUS*, with a strong expression in cell division and elongation zones of *Arabidopsis* root meristems (Luschnig et al. 1998). After 6 days of SMF exposure, we observed an increase in GUS‐staining intensities, which extended further into the root elongation and differentiation zone when compared to mock‐treated controls (Figure 3B). This argues for limited SMF‐induced alterations in auxin distribution and/or signaling in the root meristem.

Intercellular auxin transport in the primary root tip is controlled by both PIN‐FORMED (PIN) auxin efflux and AUXIN1/LIKE AUX1 (AUX1/LAX) influx carriers (Luschnig and Friml 2024). We hypothesized that subcellular localization, expression, or activity of these proteins might be affected by SMF, which in turn could affect auxin distribution and root elongation. To investigate this hypothesis, we employed mutant lines well characterized for their deficiencies in PAT and compared their overall primary root length after 6 days of SMF incubation to non‐exposed controls. Root length of the root agravitropic *aux1‐7* loss‐of‐function allele, affected in the *AUX1* auxin uptake permease (Bennett et al. 1996), did not exhibit significant differences in root length when comparing controls and SMF‐incubated seedlings (Figure 3C). This is also true for the *pin3‐5 pin4‐3 pin7‐1* triple mutant, impaired in auxin distribution in the *Arabidopsis* root tip and exhibiting defects in overall plant development (Bennett et al. 1996; Blilou et al. 2005; Dubreuil et al. 2018). This implies that cellular auxin uptake and efflux transport activities are involved in mediating MF effects on root growth (Figure 3C).

Control of root elongation is modulated by shootward auxin transport from the root tip into the root meristem elongation zone. We therefore tested for MF effects on *eir1‐1* and *eir1‐4* seedlings, both deficient in the function of the *PIN2* auxin efflux carrier, which mediates shootward auxin transport in root meristems (Luschnig et al. 1998; Abas et al. 2006). When germinated and grown for 6 days, both alleles produced primary roots, not significantly different when comparing control and SMF conditions, implying that SMFs act on shootward auxin transport in root meristems (Figure 3C). To rule out potential effects unrelated to the loss of *PIN2* function in the *eir1* alleles, we tested *eir1‐4 PIN2::PIN2:VEN*, in which loss of endogenous *PIN2* is complemented by a PIN2‐Venus translational fusion protein (Leitner et al. 2012). When cultivated under SMF conditions, this line responded with reduced root elongation, when compared to non‐treated controls, resembling root phenotypes of wild type seedlings. The apparent restoration of root phenotypes underlines a role of *PIN2* in mediating SMF effects on root elongation, substantiating a scenario in which SMFs impact coordinated PAT (Figure 3C).

### 3.3 Extended Exposure to SMFs Coincides With Altered PIN2 Protein Distribution

**FIGURE 4:**
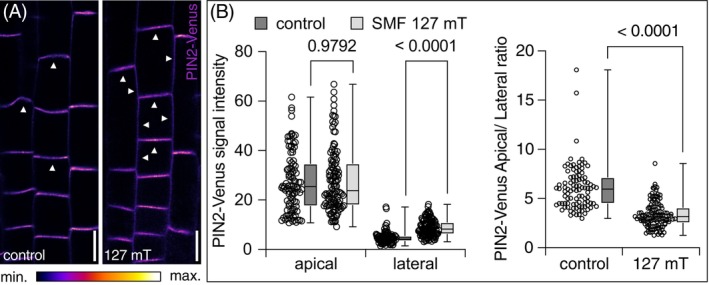
Expression and subcellular distribution of PIN2‐Venus in root meristem epidermis cells exposed to SMFs (127 mT). (A) Signals in 6‐day‐old *eir1‐4 PIN2::PIN2:VEN* root meristem epidermis cells under control conditions (left panel) and after exposure to SMF conditions (right panel). White arrowheads: Plasma membrane localization of PIN2‐Venus signals. (B) PIN2‐Venus signal intensities at the apical and the lateral domains of epidermis cells in the transition zone of root meristems of 6‐day‐old *eir1‐4 PIN2::PIN2:VEN* controls or after continuous SMF exposure (left panel). These values were used to determine apical‐to‐lateral PIN2‐Venus signal ratios in the same cells (right panel). Plasma membrane‐located signal intensities of 97 control (10 roots) and 120 SMF‐exposed cells (13 roots) were determined and normalized to grey values of the cell's interior, followed by two‐tailed t‐test analysis. Second/third quartiles and *p*‐values are indicated. Size bars: (A) = 10 μm.

The directional shootward transport of auxin depends on the polar, apical localization of PIN2 at the plasma membrane of root meristem epidermis cells (Dhonukshe et al. 2010). In light of the MF effects on *Arabidopsis* root elongation, together with the reduced MF responsiveness of *pin2* loss‐of‐function alleles, we investigated the subcellular localization of the PIN2‐Venus reporter protein after exposure to SMF conditions for 6 days. When quantifying Venus signals in *eir1‐4 PIN2::PIN2:VEN*, we did not detect any alterations in signal intensities at the apical plasma membrane domain of root meristem epidermis cells of SMF‐treated seedlings, suggesting that PIN2 expression and/or stability are unaffected (Figure 4A,B). Next, we determined the ratio of Venus signals at the apical and lateral plasma membrane domains of root meristem epidermis cells after SMF exposure. Here, we observed an increase in the relative signal intensity at the lateral plasma membrane domains when compared to mock‐treated controls (Figure 4A,B). We furthermore asked if such altered PIN2‐Venus signal distribution is reversible and allowed *eir1‐4 PIN2::PIN2:VEN* seedlings to recover from SMF conditions. Two hours after removal from the ferrite magnet slabs, we did not detect any significant differences in PIN2‐Venus signal distribution when compared to samples that remained exposed to the SMF (127 mT). After 6 h of recovery, we observed an increase in the apical/lateral signal ratio, indicative of a gradual reversion of SMF effects on PIN2‐Venus distribution (Figure S1).

Together, the alteration in PIN2‐Venus signal distribution hints at modifications in the intracellular sorting of PIN2, with strong SMFs possibly affecting lateral diffusion, accumulation in PM microdomains, or endocytic sorting from the plasma membrane, thereby compromising PIN2 polarity. As a conceivable consequence, both the directional flow and efficiency of polar auxin transport could be affected.

### 3.4 Gradient MFs Modulate Root Curvature and Auxin Distribution in *Arabidopsis* Root Meristems

Steep gradients in MFs were demonstrated to influence root curvature (Kuznetsov and Hasenstein 1996), but an involvement of auxin distribution in such responses remained to be determined. When determining gravitropic root bending under conditions of MF exposure, we did not detect any striking differences compared to control seedlings (Figure S2). Therefore, and to test for consequences of extended MF exposure on directional root growth, we designed an alternative experimental setup, incorporating a Gradient MF on a clinostat. Clinostats have long been utilized to minimize the influence of the gravitropic responses in various studies exploring plant development and responses to directional growth stimuli (Kiss et al. 1989). We employed a clinostat to simplify the analysis of crosstalk between directional root growth, the auxin transport machinery, and MFs (Figure 1B).

**FIGURE 5:**
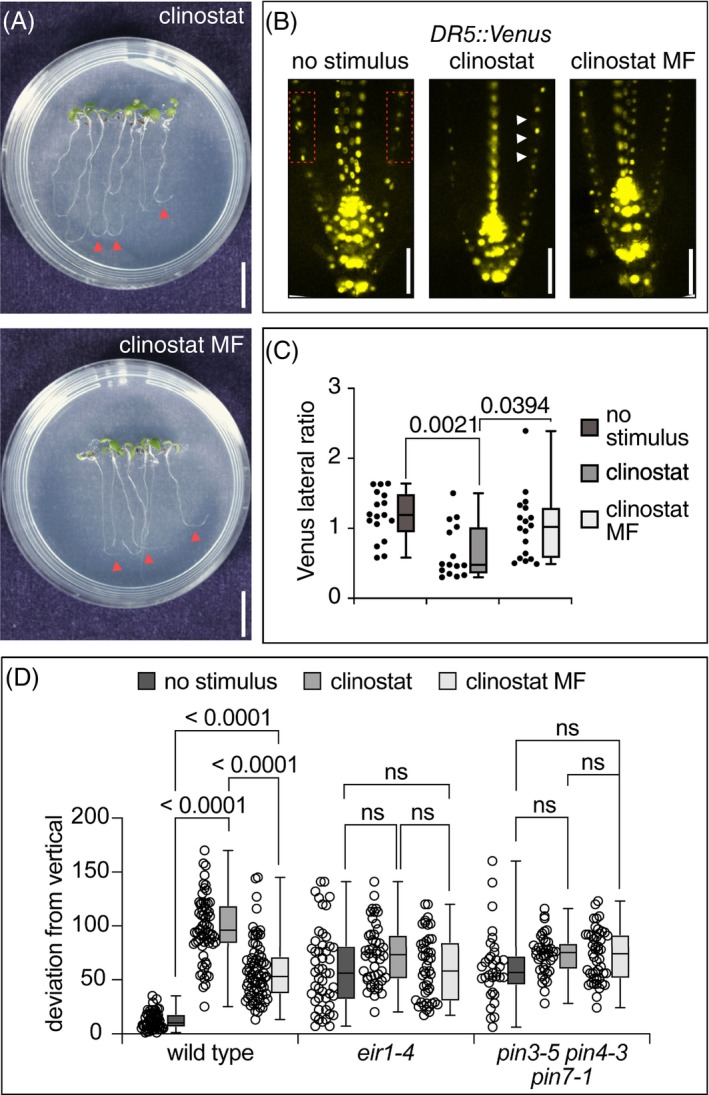
Root bending and expression of an auxin reporter in clinorotated seedlings exposed to a Gradient MF. (A) 5‐day‐old wild type seedlings after 24 h of rotation on a clinostat in the dark under control (top panels) and upon exposure to a Gradient MF (bottom panels). Arrowheads: Sites of root bending induced by clinorotation. (B) Expression of *DR5rev::3XVENUS‐N7* in 6‐day‐old controls (left), after clinorotation (middle) and after clinorotation upon exposure to a Gradient MF (right). Red rectangles: Lateral domains used for signal quantification. Arrowheads: Asymmetry in signal intensities (C) Lateral ratio of *DR5rev::3XVENUS‐N7* signals—see (B)—in 6‐day‐old control (*n* = 29), after clinorotation (*n* = 24) and after clinorotation upon exposure to a Gradient MF (*n* = 30). Lateral signal ratios obtained were then subject to one‐way ANOVA and Tukey's HSD post hoc test. *p*‐values are indicated. (D) Angle of deviation from vertical root growth in 5‐day‐old wild type, *eir1‐4* and *pin3‐5 pin4‐3 pin7‐1* seedlings, on vertically oriented control plates (control), subject to 24 h clinorotation (clinostat) or after clinorotation upon exposure to a Gradient MF (clinostat MF). A total of 34 to 74 seedlings was analyzed for each condition. Root angles are expressed as deviation from vertical (= 0°). One‐Way ANOVA and Tukey HSD were used for statistics. Second/third quartiles and *p*‐values are indicated; ns = not significant. Size bars: (A) = 5 mm, (B) = 20 μm.

A report by Su et al. (2023) summarized the consequences of low‐speed clinorotation of *Arabidopsis* seedlings, which induced directional root curvature. This was found to coincide with limited asymmetries in the expression of an auxin‐responsive reporter gene and of PIN3‐GFP, linking clinorotation‐induced root curvature to adjustments in polar auxin transport (Su et al. 2023). When positioning 5‐day‐old Col‐0 seedlings on the clinostat (on nutrient plates with the roots oriented towards the rotation axis), we detected pronounced root curvature after 24 h of incubation in darkness (Figure 5A). We furthermore detected differential expression of auxin‐responsive *DR5rev::3XVENUS‐N7* in root meristems of seedlings rotated on the clinostat for 5–6 h, implying that—analogous to gravitropic root bending—asymmetric auxin distribution defines the directionality of root growth on the clinostat (Figure 5B). We then tested for crosstalk between clinostat‐induced root curvature and directional auxin transport by analyzing auxin transport mutants. These experiments revealed only minor consequences on *eir1‐4* and *pin3‐5 pin4‐3 pin7‐1* root curvature upon incubation on the clinostat, when compared to non‐clinorotated control seedlings (Figure 5D), consistent with the findings made by Su et al. (2023) and indicating that loss of key elements of the PAT machinery renders roots less responsive to clinorotation.

For generating MF gradients, we positioned two cylindrical permanent magnets in South pole‐to‐South pole orientation and sandwiched small nutrient plates between these magnets (Zablotskii et al. 2016). This setup generates a Gradient MF, resembling conditions previously demonstrated to impact root curvature (Figure 1B–D) (Hasenstein et al. 2013, 2022). Roots of 6‐day‐old seedlings germinated on these nutrient plates were positioned exactly between the magnets and subjected to clinorotation in the darkness for 24 h. As controls, we used a similar experimental setup but lacking the magnets. When scoring root curvature of wild‐type seedlings, we detected a reduction in root curvature in seedlings exposed to Gradient MF vs. controls (Figure 5A,D). Furthermore, analysis of *DR5rev::3XVENUS‐N7* expression revealed that Gradient MF conditions disrupt the formation of an expression gradient, suggesting that MF exposure interferes with root curvature via auxin‐derived signals (Figure 5B,C). Consistent with this hypothesis, the root curvature of 6‐day‐old clinorotated *eir1‐4* and *pin3‐5 pin4‐3 pin7‐1* auxin transport mutants exposed to Gradient MFs did not differ significantly from the root curvature of controls (Figure 5D).

Overall, Gradient MF exposure under conditions of a diminished influence of the gravity vector revealed suppression of root curvature. This inhibitory effect seemingly involves crosstalk between Gradient MF conditions and elements of the PAT machinery.

### 3.5 Gradient MFs Antagonize Lateral PIN3‐YFP Distribution in *Arabidopsis* Root Meristems

The reduced responsiveness of *eir1‐4* and *pin3‐5 pin4‐3 pin7‐1* roots to clinorotation and to exposure to Gradient MFs hints at alterations in auxin homeostasis via modulation of the plant's auxin transport machinery. We therefore tested for Gradient MF effects on PINs by determination of PIN2‐Venus and PIN3‐YFP reporter protein distribution in 6‐day‐old clinorotated *eir1‐4 PIN2::PIN2:VEN* and *PIN3::PIN3:YFP* seedlings.

**FIGURE 6:**
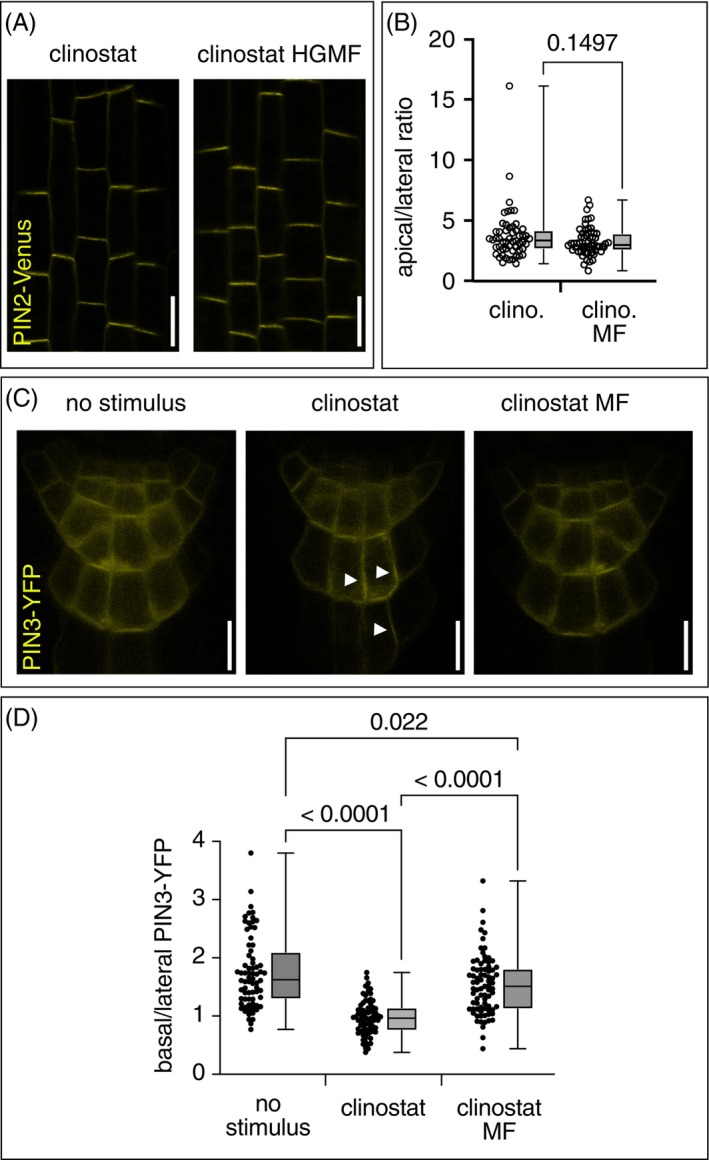
Subcellular PIN localization in root meristems exposed to Gradient MFs. (A) PIN2‐Venus signal localization at the plasma membrane of root meristem epidermis cells of 6‐day‐old *eir1‐4 PIN2::PIN2:VEN* clinorated for 90–120 min (left) or clinorotated upon exposure to a Gradient MF (right). (B) Quantification of PIN2‐Venus apical/lateral signal ratios in root meristem epidermis cells of clinorotated (*n* = 54) and clinorotated/Gradient MF‐exposed seedlings (*n* = 59)—as described in (A). (C) PIN3‐YFP signal distribution in root cap columella cells in 6‐day‐old vertically oriented seedlings (no stimulus) after 90–120 min of clinorotation (clinostat) and after clinorotation but exposed to a Gradient MF (clinostat MF). Arrowheads indicate lateral accumulation of YFP signals. (D) Quantification of basal/lateral PIN3 signal distribution in root cap columella cells, under conditions as described under (C). Signals in 72 to 79 root cap columella cells were quantified for each condition. Fifteen to nineteen root caps were analyzed in two biological replicates. One‐Way ANOVA and Tukey HSD were used for statistics. Second/third quartiles and *p*‐values are indicated. Size bars: (A, C) = 10 μm.

After 90–120 min of clinorotation under Gradient MF conditions, no significant adjustments in PIN2‐Venus signal distribution were observed when compared to clinorotated controls (Figure 6A,B). Thus, different from long‐term exposure to SMFs, short exposure to Gradient MF has no evident consequences for PIN2 distribution. Clinorotation of *PIN3::PIN3:YFP* for 90–120 min produced lateralization of PIN3‐YFP signals in columella root cap cells when compared to non‐stimulated controls (Figure 6C,D). This shows that root curvature and differential expression of auxin‐responsive *DR5rev::3XVENUS‐N7* in clinorotated roots (Figure 5B,C) coincide with polar PIN3 distribution in root cap cells. Remarkably, when scoring PIN3‐YFP polarity in clinorotated roots that were exposed to Gradient MF conditions, lateral reporter signal accumulation was significantly less prominent than in clinorotated samples but rather resembled the distribution in non‐clinorotated controls (Figure 6C,D). Since statolith displacement in columella root cap cells has been intimately linked to redirecting auxin flow and PIN distribution (Furutani et al. 2020), we asked if similar responses might guide PIN3‐YFP localization upon exposure to Gradient MF conditions. For this, we employed 5‐day‐old wild‐type seedlings and compared statolith distribution in vertically positioned seedlings after 2 h of clinorotation and after 2 h of clinorotation and exposure to Gradient MF conditions. Consistent with earlier observations (Villacampa et al. 2021), clinorotation produced a more dispersed distribution of statoliths when compared to controls, which exhibit statolith accumulation at the bottom of columella root cap cells (Figure S3). A similar dispersed statolith distribution was observed in clinorotated seedlings exposed to Gradient MF conditions (Figure S3), demonstrating limited consequences of Gradient MF exposure on statolith distribution under our experimental conditions.

When taken together, our findings indicate a dampening effect of Gradient MF exposure on PIN3 polarization, consistent with Gradient MFs acting on root curvature via control of intracellular PIN3 sorting.

## 4 Discussion

Plant growth is defined by environmental parameters, amongst which the GMF remained largely unnoticed (Maffei 2014; Dhiman and Galland 2018; Sarraf et al. 2020). Therefore, a deeper understanding of molecular mechanisms underlying MF effects seems desirable. Our findings provide insights into cellular adjustments triggered by prolonged exposure to an SMF or caused by a Gradient MF, both impacting vital plant responses via elements of the auxin transport machinery.

Crosstalk between plant growth regulators, such as auxin, and MFs has meanwhile been firmly established. Selected *PIN* genes and *Aux/IAA* genes, for example, were found to be upregulated under NNMF conditions (Xu et al. 2018). Furthermore, loci involved in IAA metabolism, such as *TAA1, YUC4*, and *GH3.9*, were upregulated following 200 mT SMF exposure (Zhou et al. 2024). The same report also demonstrated upregulation of *LAX2, PIN6*, and several *Aux/IAA* genes in *Arabidopsis* roots and/or shoots, further highlighting a relationship between SMF‐mediated growth adjustments and auxin, yet modes of such crosstalk remained unknown (Zhou et al. 2024). A link between directional auxin transport and its adjustment in response to MFs has been established by Jin et al. (2019) in *Arabidopsis* seedlings. Their setup employed 600 mT MFs and caused root growth promotion of *Arabidopsis* seedlings. Control of auxin distribution appears to be involved in this growth response, as the abundance of AUX1 and PIN3 reporter proteins was affected by MF exposure, whereas root growth of mutants deficient in the corresponding genes turned out to be less responsive to SMFs (Jin et al. 2019). We also detected reduced MF responsiveness of mutant seedlings affected in auxin transport when employing our SMF setup (Figure 1A). However, when quantifying apical PIN2‐Venus signal intensities, no prominent alterations in overall signal intensities could be observed. Instead, we found that the apical‐to‐lateral ratio of PIN2‐Venus signals is compromised, reflected in an increase in lateral PIN2‐Venus signals upon SMF exposure. In light of the rate‐limiting role attributed to the dynamics of PIN plasma membrane distribution in the control of PAT, such PIN2 lateralization could affect spatial auxin distribution. This would be consistent with the increased *AtIAA2::GUS* activity in root meristems exposed to SMFs and with the diminished MF‐induced growth responses observed with *pin2* alleles.

An auxin transport reflux loop has been put forward as a central element in the regulation of root growth and patterning. In simple terms, this pathway involves shootward auxin transport from the root tip into the root meristem transition/elongation zone via the outermost meristem cell layers, followed by rootward auxin recycling to the root tip via ground tissue and vasculature. Disturbances of such auxin reflux by manipulating the function of auxin transport proteins involved have been demonstrated to feedback on the control of root morphogenesis (Blilou et al. 2005; Marhavy et al. 2014). It remains to be determined if SMF‐mediated adjustments in PIN2 polarity could affect directional auxin transport and root growth in a similar fashion. This seems even more relevant as another report described contrasting *Arabidopsis* root growth responses to MF conditions (Jin et al. 2019). Strikingly, Jin and colleagues observed enhanced *Arabidopsis* root elongation, whereas our conditions produced an inhibitory effect.

It needs to be emphasized that Jin et al. (2019) exposed their *Arabidopsis* seedlings closely attached to the side of a single strong magnet (600 mT), whereas our two‐magnet approach established substantially weaker MFs (127 mT and less). Strikingly, Jin and colleagues also found that adjustments in the orientation of the MF cause variable plant growth responses. In their settings, positioning seedlings in a MF parallel or perpendicular to the gravity vector resulted in enhanced root elongation, whereas the same setup with the MF oriented opposite to the gravity vector failed to stimulate root elongation (Jin et al. 2019). Thus, apart from differing responses observed upon exposure to MFs of distinct field strength, modifications in the orientation of the MF lines cause variable growth responses, even under otherwise identical experimental conditions. It is tempting to speculate about the mechanisms by which variations in the orientation of MFs influence transcriptional as well as post‐transcriptional control of elements of the auxin transport machinery. This might involve, so far unspecified, crosstalk between strong MFs and gravitational forces. However, at this stage, the nature of such processes remains entirely enigmatic.

The disturbance of directional root growth responses that we observed in a Gradient MF further highlights the diversity of plant growth responses induced by MFs. Gradient MFs exert a force through diamagnetic susceptibility of the exposed objects, generating plant responses that might even overrule gravity effects. Earlier work employing HGMFs in close proximity to magnetized wedges demonstrated intracellular magnetophoresis of amyloplasts in columella root cap cells, together with HGMF‐induced root curvature (Kuznetsov and Hasenstein 1996). Thus, by analogy to root gravitropism, statolith displacement might function as a trigger for directional root curvature toward HGMFs (Hasenstein et al. 2013). In our modified approach, we employed less steep MF gradients in combination with seedlings grown on a clinostat, suitable for studying MF effects over extended periods of time. Low‐speed clinorotation, as used in our experiments, has been demonstrated to induce root curvature, possibly enhanced by the seedlings' gravistimulation during the initial stages of clinorotation (Su et al. 2023). Consistent with a role for auxin, clinorotated seedlings did establish a lateral gradient in the expression of an auxin‐responsive reporter protein, whilst mutants deficient in directional auxin transport failed to respond with uniform root curvature. Furthermore, and comparable to the situation in gravity‐responding roots, asymmetry in the expression of PIN3‐GFP detected in clinorotated columella root cap cells could define an auxin gradient responsible for root curvature (Su et al. 2023).

Our observations further corroborate this model, as we could demonstrate that clinorotation provides sufficient directional information for the induction of root curvature that coincided with asymmetries in the expression of an auxin reporter and resulted in increased PIN3‐YFP polarization in columella root cap cells. Seedling exposure to Gradient MFs, however, moderates the effects of clinorotation, with such seedlings behaving more like non‐clinorotated controls. This is reflected in diminished root curvature and reduced PIN3‐YFP lateralization in columella root cap cells, which perhaps negatively feeds back on the establishment of a lateral auxin gradient. In light of results obtained in earlier experiments, in which HGMF exposure has been demonstrated to impact statolith localization in columella root cap cells (Kuznetsov and Hasenstein 1996), it appeared plausible that Gradient MF exposure, as employed in our experiments, exerts similar effects on these determinants of gravity perception. Our analysis of amyloplast distribution on a clinostat indeed argues for clinorotation‐induced displacement, which is consistent with earlier observations (Villacampa et al. 2021). Nevertheless, the application of a Gradient MF did not cause any further prominent alterations in amyloplast distribution, arguing for comparably subtle MF effects when compared to those described in earlier experiments (Kuznetsov and Hasenstein 1996). Furthermore, rapid physiological responses preceding statolith sedimentation and implying the involvement of additional signaling mechanisms (Muthert et al. 2019) might contribute to PIN3‐YFP localization control under Gradient MF conditions.

The dynamics in the subcellular distribution of PINs is essential for various aspects of plant morphogenesis as well as adaptive growth responses and has been studied extensively (Luschnig and Friml 2024). Sorting of PIN2, in particular, involves polarized exocytic sorting to the apical domain of root meristem epidermis cells. This is followed by lateral diffusion within the plasma membrane bilayer and subsequent endocytic sorting via Clathrin‐Mediated Endocytosis (CME). The sites of PIN2 CME are preferentially located at the outermost margins of the apical plasma membrane domain, thereby restricting the vast majority of the plasma membrane‐localized PIN2 fraction to this domain (Kleine‐Vehn et al. 2011). In addition, a subfraction of endocytosed PIN2 is subject to ubiquitination and subsequent sorting to the lytic vacuole for its proteolytic degradation, further impacting PIN2 abundance at the plasma membrane (Leitner et al. 2012). Determination of PIN2‐Venus steady‐state signals upon seedling exposure to SMF conditions did not hint at any striking differences in PIN2 levels at the apical plasma membrane domain. Nevertheless, PIN2 distribution appears less restricted to the apical domain, signified by elevated PIN2‐Venus signals at the lateral plasma membrane domains. This would indicate that, under SMF conditions, either PIN2 lateral diffusion or CME is affected, leading to ectopic accumulation of PIN2 outside of its designated plasma membrane domain. As for PIN3 in root tips, gravity‐induced polarization at lateral plasma membrane domains is guided by CME and depends on guanine nucleotide exchange factors for ARF GTPase (ARF‐GEF) cargo sorting determinants. As a result, PIN3 gets rerouted to selectively enrich at polar plasma membrane domains by means of protein transcytosis (Kleine‐Vehn et al. 2010). Similar to PIN2 polarity under SMF conditions in root epidermis cells, PIN3 polarity acquisition in root cap columella cells is affected upon exposure to Gradient MFs, potentially as a consequence of altered protein sorting under these particular conditions.

Our findings suggest that MFs trigger comparably fast responses in protein polarity control, as is the case for PIN3 in clinorotated root cap cells, as well as effects that become apparent after extended exposure to MF conditions, as observed for PIN2 in the root epidermis. It remains to be resolved how these responses are brought about in mechanistic terms. Recent studies have underscored a crucial impact of external mechanical and magnetic forces on cell shape, function, and fate through their physical interactions with the cytoskeletal network (Zablotskii et al. 2016). Furthermore, assessment of the barley root and shoot proteomes upon exposure to MFs revealed changes in the abundance of a range of proteins, amongst which actin and components of Coat Protein Complexes and the Clathrin Coat were identified (Shabrangy et al. 2021). Whilst all these proteins do exert distinct functions in intracellular protein trafficking, it remains to be determined if such altered protein abundance could result in any noticeable impact on intracellular sorting processes and how such adjustments could affect the fate of PIN proteins.

## Author Contributions

A.S. and C.L. planned and designed the research. A.S. performed experiments and data analysis. A.S. and C.L. wrote the manuscript.

## Supporting information


**Data S1.** Supporting Information Figure.

**Data S2.** Supporting Information.

## Data Availability

The data presented in this publication were collected from lab experiments. The authors included all the results in this work, with crude datasets available as Supporting Information. The physical data that support the findings of this study are available from the corresponding first author (A.S.) upon reasonable request.
