# Supplementary material for: Magnetic Fields Impact on PIN‐FORMED Protein Polarity in Arabidopsis thaliana
Source: Physiol Plant. 2025 May 23;177(3):e70274. doi: 10.1111/ppl.70274 (PMC12100457; doi:10.1111/ppl.70274)
Supplement: Supplementary file 1 — Data S1. Supporting Information Figure. [file PPL-177-e70274-s002.pdf]

Magnetic fields impact on PIN-FORMED protein polarity in *Arabidopsis thaliana*

Azita Shabrangy*[1] & Christian Luschnig[2]

1) Molecular Systems Biology (MOSYS), Department of Functional and Evolutionary Ecology, University of Vienna, Vienna, Austria

2) Institute of Molecular Plant Biology (IMPB), BOKU University, Muthgasse 18, 1190 Wien, Austria

* Correspondence to: azita.shabrangy@univie.ac.at

Supplementary Figures

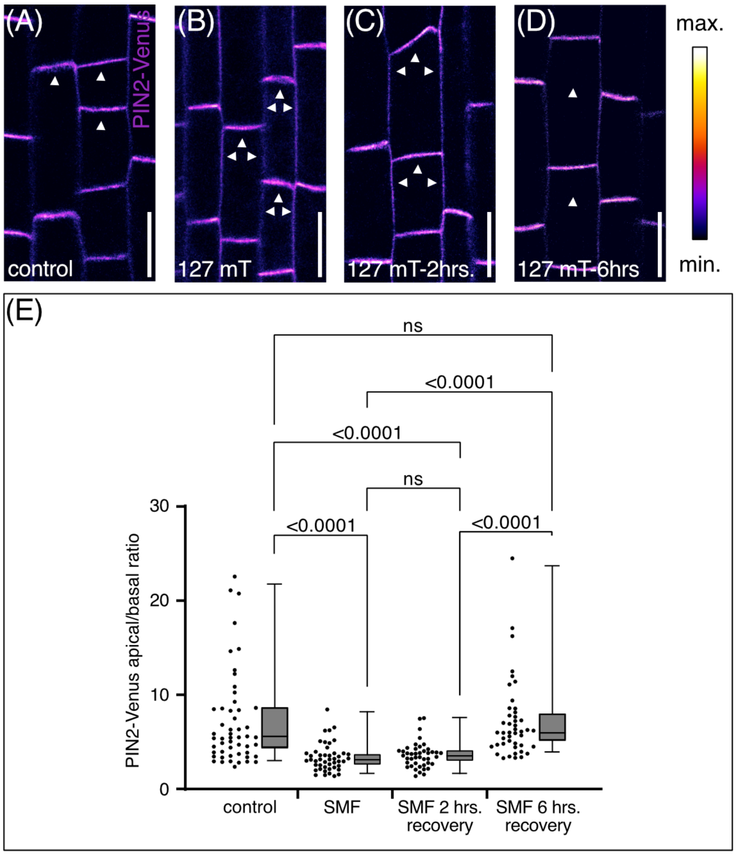

Figure S1. Redistribution of PIN2-Venus signals in *eir1-4 PIN2::PIN2:VEN* root meristem epidermis cells, after recovery from SMF conditions. (A) Venus signals in root epidermis cells of 6-day-old control seedlings flanked by wooden blocks. (B) Venus signals in root epidermis cells of 6-day-old seedlings flanked by ferrite magnets producing a field of 127 mT. (C) Venus signals in root epidermis cells of 6-day-old seedlings flanked by ferrite magnets producing a field of 127 mT followed by 2 hours recovery from SMF conditions. (D) Venus signals in root epidermis cells of 6-day-old seedlings flanked by ferrite magnets producing a field of 127 mT followed by 6 hours recovery from SMF conditions. (E) PIN2-Venus signal intensities at the apical and the lateral domains of epidermis cells in the transition zone of root meristems of 6-day-old *eir1-4 PIN2::PIN2:VEN* controls, after continuous SMF exposure (127 mT) as well as after a recovery phase of 2 and 6 hours, respectively. Values were used to determine apical/lateral PIN2-Venus signal ratios in individual cells. Plasma membrane-located signal apical/lateral ratios of control seedlings (n = 52, 6 roots), SMF-exposed seedlings (n = 46, 5 roots), SMF-exposed seedlings followed by 2 hours recovery (n = 43, 6 roots) and SMF-exposed seedlings followed by 6 hours recovery (n = 45, 6 roots) were determined, followed by one-way ANOVA and Tukey's HSD post hoc test. Second/third quartiles and p-values are indicated; ns = not significant. Size bars: (A-D) = 10 μm.

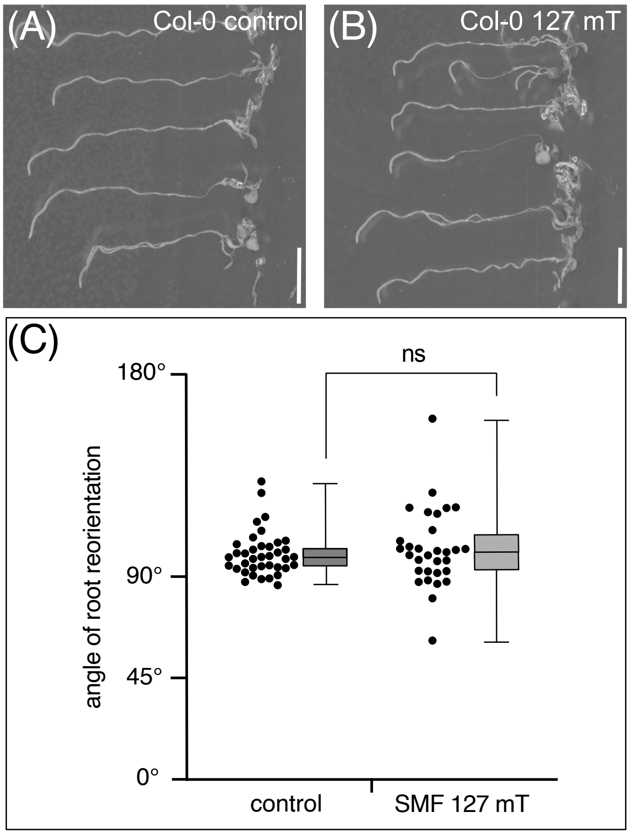

Figure S2. Gravitropic root growth is not visibly affected upon exposure to SMF conditions. (A,B) *Arabidopsis* wild type Col-0 seedlings were germinated on PNS, followed by transfer to fresh nutrient plates after 5 days. Control seedlings were positioned between wooden blocks (A), whereas SMF-exposed seedlings were positioned between two ferrite magnets producing a field of 127 mT (B). After 24 hours, the plates were turned clockwise at an angle of 90°, followed by determination of gravitropic root bending after o/n incubation. (C) Reorientation of root growth in control seedlings (n = 38) and in seedlings exposed to SMF (n = 31), after o/n gravistimulation was determined, followed by two-tailed t-test. Second/third quartiles and the p-value are indicated; ns = not significant. Size bars: (A,B) = 5 mm.

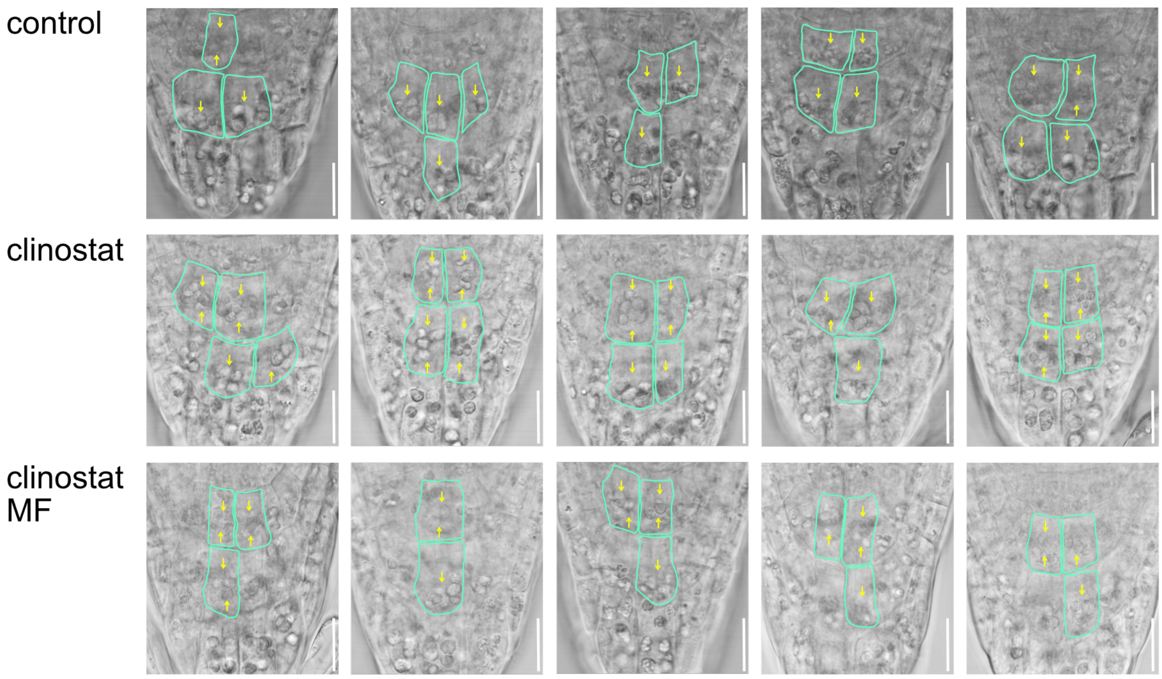

Figure S3. Subcellular localization of statoliths in root cap columella cells of 5-day-old Arabidopsis seedlings. Top row: vertically grown control; middle row: after 2 hours rotation on the clinostat; bottom row: after 2 hours rotation on the clinostat exposed to a Gradient MF. Arrows: accumulation of statoliths at the bottom (arrows pointing downwards); intermediate (arrows pointing up- and downwards); upper half of statocytes (arrows pointing upwards). Cell boundaries of statocytes are outlined. Size bars: = 10 µm.
